# Evidence of Divergent Amino Acid Usage in Comparative Analyses of R5- and X4-Associated HIV-1 Vpr Sequences

**DOI:** 10.1155/2017/4081585

**Published:** 2017-05-17

**Authors:** Gregory C. Antell, Will Dampier, Benjamas Aiamkitsumrit, Michael R. Nonnemacher, Vanessa Pirrone, Wen Zhong, Katherine Kercher, Shendra Passic, Jean Williams, Yucheng Liu, Tony James, Jeffrey M. Jacobson, Zsofia Szep, Brian Wigdahl, Fred C. Krebs

**Affiliations:** ^1^Department of Microbiology and Immunology, Drexel University College of Medicine, Philadelphia, PA, USA; ^2^Center for Molecular Virology and Translational Neuroscience, Institute for Molecular Medicine and Infectious Disease, Drexel University College of Medicine, Philadelphia, PA, USA; ^3^School of Biomedical Engineering, Science, and Health Systems, Drexel University, Philadelphia, PA, USA; ^4^Center for Clinical and Translational Medicine, Institute for Molecular Medicine and Infectious Disease, Drexel University College of Medicine, Philadelphia, PA, USA; ^5^Division of Infectious Diseases and HIV Medicine, Department of Medicine, Drexel University College of Medicine, Philadelphia, PA, USA; ^6^Sidney Kimmel Cancer Center, Thomas Jefferson University, Philadelphia, PA, USA

## Abstract

Vpr is an HIV-1 accessory protein that plays numerous roles during viral replication, and some of which are cell type dependent. To test the hypothesis that HIV-1 tropism extends beyond the envelope into the *vpr* gene, studies were performed to identify the associations between coreceptor usage and Vpr variation in HIV-1-infected patients. Colinear HIV-1 Env-V3 and Vpr amino acid sequences were obtained from the LANL HIV-1 sequence database and from well-suppressed patients in the Drexel/Temple Medicine CNS AIDS Research and Eradication Study (CARES) Cohort. Genotypic classification of Env-V3 sequences as X4 (CXCR4-utilizing) or R5 (CCR5-utilizing) was used to group colinear Vpr sequences. To reveal the sequences associated with a specific coreceptor usage genotype, Vpr amino acid sequences were assessed for amino acid diversity and Jensen-Shannon divergence between the two groups. Five amino acid alphabets were used to comprehensively examine the impact of amino acid substitutions involving side chains with similar physiochemical properties. Positions 36, 37, 41, 89, and 96 of Vpr were characterized by statistically significant divergence across multiple alphabets when X4 and R5 sequence groups were compared. In addition, consensus amino acid switches were found at positions 37 and 41 in comparisons of the R5 and X4 sequence populations. These results suggest an evolutionary link between Vpr and gp120 in HIV-1-infected patients.

## 1. Introduction

HIV-1 entry proceeds via direct interaction between the viral envelope glycoprotein (gp) 120 and the host cell CD4 receptor molecule, as well as one of two coreceptor molecules, CCR5 or CXCR4 [1]. The HIV-1 gp120 envelope protein consists of five hypervariable regions (V1–V5) interspersed among five constant regions (C1–C5), with the third variable loop (V3) region serving as the major determinant in HIV-1 coreceptor phenotype. Specifically, V3 consists of 34–36 amino acid residues (most commonly 35 residues). However, as conformational changes within the V3 loop occur upon the binding of gp120 with CD4, it is possible that the coreceptor usage-associated amino acid residues within other regions of envelope participate in the structural rearrangement of gp120 [2, 3]. Patients with HIV-1 infections that are not well suppressed by effective antiretroviral therapy will experience some form of coreceptor switch, in which CXCR4-utilizing (X4) viruses gradually emerge from a predominantly CCR5-utilizing (R5) swarm due to the accumulation of amino acid changes within the V3 loop, particularly at amino acid positions 11 and 25. HIV-1 tropism based on coreceptor usage was previously used interchangeably with tropism defined by cellular target. However, recent studies have concluded that it is more precise to discuss viral infection in terms of utilization of a coreceptor alongside the phenotype of the target cell [4, 5]. For genomic applications, coreceptor utilization prediction algorithms can be effectively used to classify Env-V3 sequences computationally, as previously reviewed [6].

Variations in Env-V3 sequences that alter coreceptor phenotype are specific examples of sequence changes that take place throughout the HIV-1 genome during an ongoing HIV-1 replication. The present studies were based on the general hypothesis that changes in Env-V3 that alter the coreceptor phenotype lead to changes elsewhere in the genome. This is likely due to functional changes that are linked to coreceptor usage and, by extension, the identity of the infected cell. The overall goal of this study was to perform comparative analyses of the accessory viral protein R (Vpr) with regard to R5 and X4 HIV-1 quasispecies. Vpr is a 96 amino acid protein with three characteristic *α*-helical secondary structures, *α*-helix 1 (positions 17–33), *α*-helix 2 (positions 38–50), and *α*-helix 3 (positions 53–78). The three helices are flanked by two, nonstructural, but functional, N- and C-terminal domains [7–9]. In general, Vpr has been understudied relative to other HIV-1 proteins, despite its multifaceted roles in the replication cycle, activity as an extracellular protein, and contributions to HIV-1-associated neuropathogenesis. Most critically, Vpr is essential for viral replication in macrophages but dispensable in T cells, suggesting cell type-specific functionality and possible coevolution with Env-V3 [10]. Since coreceptor usage is an important (but not absolute) determinant of cellular tropism, we hypothesized that coreceptor-associated genetic signatures would emerge in Vpr due to cell type-specific selective pressures that similarly direct coreceptor usage. Support for this hypothesis was provided by previous reports of specific HIV-1 Tat and LTR sequence signatures associated with coreceptor utilization, suggesting that selective pressures applied within specific cellular targets during the course of HIV-1 infection result in distinct transcriptional regulatory mechanisms [3, 6, 11]. Although coreceptor usage has been clearly linked to HIV-1 transmission as well as HIV-1-associated disease progression [12–15], the degree and specificity of variations in Vpr that might contribute to such clinically significant events remain unexplored.

In this study, we extended a genetic approach previously applied to investigate gp120, Tat, and LTR associations with coreceptor utilization [3] to the accessory protein Vpr. Aligned Vpr sequences were grouped as X4 or R5 according to the predicted coreceptor utilization of the colinear V3 region and evaluated independently at each Vpr amino acid position with regard to amino acid diversity and Jensen-Shannon divergence between the groups. Combined diversity and divergence metrics permitted the quantitative characterization of position-specific amino acid variation and provided a methodology for comparing sequence information between different populations. Overall, this strategy provided a concise genetic approach that revealed specific amino acid positions and side chain motifs in Vpr that were coselected with CXCR4 or CCR5 coreceptor utilization during replication in HIV-1-infected patients.

## 2. Materials and Methods

### 2.1. Collection and Sequencing of DNA from HIV-1-Infected Patients

Patients enrolled in the Drexel Medicine CNS AIDS Research and Eradication Study (CARES) Cohort were recruited from the Partnership Comprehensive Care Practice of the Division of Infectious Diseases and HIV Medicine in the Department of Medicine at Drexel University College of Medicine (Philadelphia, Pennsylvania, USA) and the Center for Clinical and Translational Medicine in the Institute for Molecular Medicine and Infectious Disease. Patients in the Drexel Medicine CARES Cohort were recruited under protocol 1201000748 (Brian Wigdahl, PI), which was approved by the Drexel University College of Medicine Institutional Review Board (IRB) and adheres to the ethical standards of the Helsinki Declaration (1964, amended most recently in 2008) developed by the World Medical Association. All patient samples were collected under the auspices of this protocol through written consent obtained upon patient enrollment. Whole blood collected from each patient was processed and assessed as previously described [16]. Peripheral blood samples were used for drug screening, plasma analysis, and PBMC isolation as previously described. Env-V3 and Vpr regions were sequenced from 4.4 kb fragments [11, 16].

### 2.2. Compiling Colinear HIV-1 Vpr-Env Sequences

HIV-1 sequences from the Los Alamos National Laboratory (LANL) HIV Sequence Database that included colinear *env*-V3- and *vpr*-coding regions were collected and annotated in October 2014, while additional HIV-1 sequences from patient samples collected from the Drexel Medicine CARES Cohort were included to supplement the publicly available dataset. The Drexel Medicine CARES Cohort is a predominantly subtype B patient population from Philadelphia, Pennsylvania [11, 16–18]. To reduce the impact of sequence variation due to regional differences, the query of the LANL sequence database was limited to include only North American sequences. Furthermore, the search query was restricted to one sequence per patient and excluded laboratory strain sequences or those used for previous functional studies. Vpr sequences added from the Drexel Medicine CARES Cohort have been submitted to GenBank under BioProject sub1322660.34.

### 2.3. Coreceptor Usage Classification

In silico, coreceptor usage predictions were performed using WebPSSM on Env-V3 amino acid sequences colinear with the Vpr sequences. Sequences were classified as CCR5 or CXCR4 utilizing (R5 or X4) [19]. This algorithm classifies CXCR4 coreceptor utilization at both high sensitivity (84%) and specificity (96%) [19]. Exclusion methods were applied to reduce noise introduced by WebPSSM predictions as previously discussed [20]. Specifically, V3 sequences were required to be exactly 35 amino acids in length, passed the standard quality control metrics implemented by WebPSSM, and have a WebPSSM score outside the “indeterminate range” (defined as scores between −2.88 and −6.96). With the application of this filtering method, the genetic analyses were focused on sequences with the greatest confidence classification in the PSSM-derived distribution, resulting in the definitive characterization of Env-V3 sequences as CCR5- or CXCR4-utilizing genotypes [11].

### 2.4. Reduced Amino Acid Alphabets

Reduced amino acid alphabets were utilized in order to gain deeper insight into the structural and functional consequences of amino acid substitutions. The 20-letter amino acid alphabet was reduced to smaller alphabets based on correlations indicated by the BLOSUM50 similarity matrix [21] and implemented in the Biopython version 1.66 [22]. The resulting alphabets were designated as Murphy-15, Murphy-10, Murphy-8, and Murphy-4, referring to the number of letters that make up each alphabet. Accordingly, all diversity and divergence calculations were performed on reduced amino acid alphabets in addition to the full alphabet containing 20 distinct amino acids. Whereas diversity and divergence metrics may be oversensitive to amino acid variation between amino acids with similar physiochemical properties (K versus R for example), reduced alphabets may overgeneralize and exclude important sequence information. Overall, this methodology aims to better focus on the truly important regions of a protein by observing trends in diversity and divergence across multiple alphabets.

### 2.5. Positional Diversity Analysis

Vpr sequences were aligned using Multiple Sequence Comparison by Log-Expectation (MUSCLE), (version 5.05) [23] using default parameters, with insertions and gaps removed to preserve reference numbering relative to the HXB2 reference sequence (K03455). The amino acid diversity of each position was calculated using a window length, *w*, of 1 and an order of 1 [equivalent to exp (Shannon entropy with base *e*)] according to the following equation [24]:

(1) $$\begin{array}{c} D_{w,p}=\exp\left(-\overset{R_{w,p}}{\underset{i=1}{\sum }}p_{i_{w,p}}\ln\left[p_{i_{w,p}}\right]\right). \end{array}$$

In this definition, genetic diversity, *D*, is defined as a weighted sum of all variants, *p*, at a given position, *i*, in the amino acid sequence. While any window length, *w*, may be applied, *w* = 1 was used to independently assess diversity at each position within a multiple sequence alignment. The amino acid diversity calculates the effective number of amino acids present at each alignment position and gives greater weight to neither rare nor abundant species when the order = 1. Generally, low-diversity positions have a higher likelihood of exhibiting structural and functional importance, while positions more permissive to structural and functional variation display higher diversity at the amino acid level [25].

### 2.6. Positional Jensen-Shannon Divergence Analysis

Jensen-Shannon divergence, which is based on Kullback-Leibler divergence, measures the similarity between two probability distributions. This analysis has been successfully applied to previous profile-to-profile multiple sequence alignment comparisons [26, 27]. Jensen-Shannon divergence scores are bound in the range of 0 (completely similar) and 1 (completely dissimilar), with dissimilarity identifying positions of interest. Position frequency matrices (PFMs) were generated from multiple sequence alignments (MSA) of R5- and X4-classified sequence populations, with each PFM containing the relative abundance for each potential residue (or residue group in the case of reduced alphabets) across all positions. PFMs derived from R5- and X4-classified Vpr sequences were used to compute the Jensen-Shannon divergence between populations using the following equation [3]:

(2) $$\begin{array}{c} D_{js}=\frac{1}{2}\left[\overset{20}{\underset{a=1}{\sum }}Q_{a}^{1}\log_{2}\frac{Q_{a}^{1}}{Q_{a}^{0}}+\overset{20}{\underset{a=1}{\sum }}Q_{a}^{2}\log_{2}\frac{Q_{a}^{2}}{Q_{a}^{0}}\right], \end{array}$$

where *Q*_*a*_^0^ = 1/2(*Q*_*a*_^1^ + *Q*_*a*_^2^).

### 2.7. Statistical Analysis

Statistical analyses were completed in custom IPython Notebooks using the SciPy Python library (version 0.14.0). To identify statistically the significant positions, a Monte Carlo permutation test (*n* = 1000) was applied for groups of size *M* and *N*, where *M* and *N* represent the number of sequences in the X4 and R5 Vpr groups, respectively. Numerical integration of the probability density function (PDF) of the randomly generated Jensen-Shannon divergence distribution was implemented using a Gaussian kernel density estimator in SciPy. *P* values were determined according to the probability of arriving at a random value equal to or greater than the true Jensen-Shannon divergence.

## 3. Results and Discussion

Studies of coordinated amino acid variation between Vpr and gp120 were initiated using HIV-1 DNA sequences containing both *vpr* and *env*. Sequences of clinical origin were derived from blood samples collected from HIV-1-infected patients in the Drexel Medicine CNS AIDS Research and Eradication Study (CARES) Cohort [11, 16–18, 28, 29]. Colinear DNA was isolated from patient PBMCs and amplified using primers specifically designed to encompass the Vpr- and gp120-coding sequences. Similarly, DNA sequences from the Los Alamos National Laboratory (LANL) HIV-1 Databases **(**www.hiv.lanl.gov**)** were selected based on the availability of both reading frames in a single, colinear sequence. Sequences derived from patient DNA and acquired from the LANL Databases were first analyzed to determine the putative coreceptor phenotype of the encoded gp120 glycoprotein. Sequences were analyzed using WebPSSM, an established and recognized online tool for predicting coreceptor usage from amino acids encoded by HIV-1 gp120 V3 sequences [19]. Of the 1010 colinear sequences included in the final analysis, 969 were found to contain V3 sequences that scored as R5 genotypes. In contrast, only 41 sequences were scored as X4 genotypes (Table 1). The total set of sequences analyzed included 29 samples from the Drexel CARES Cohort, 20 of which were classified as R5.

### 3.1. HIV-1 Vpr Amino Acid Diversity Is Concentrated in Two Major Regions of Vpr

**Figure 1:**
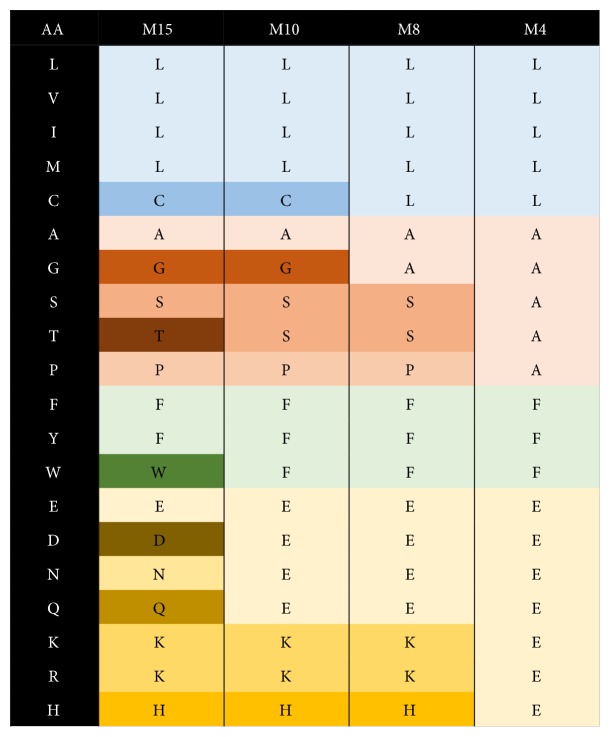
Reduced alphabets utilized for analysis of Vpr amino acid variation. The 20 standard amino acids were grouped according to BLOSUM50 correlations in a hierarchical manner as previously reported in [21]. The resulting alphabets were designated as Murphy-15, Murphy-10, Murphy-8, and Murphy-4. Colors represent amino acids grouped in each alphabet according to physiochemical properties (M15, M10, M8, and M4, resp.).

**Figure 2:**
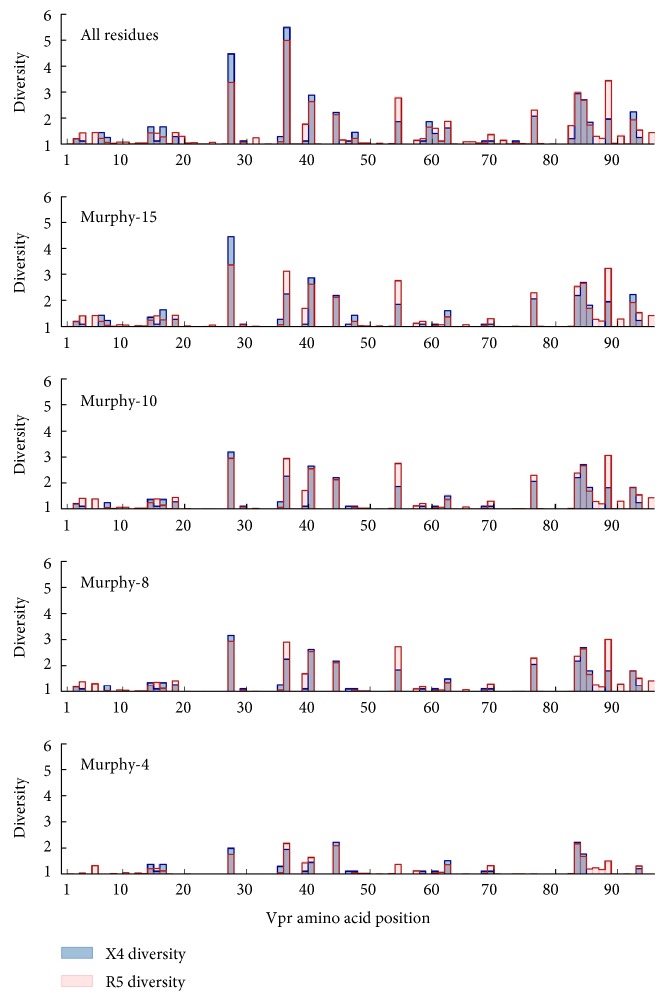
Position-specific Vpr amino acid diversity is generally consistent across multiple amino acid alphabets but different between R5- and X4-associated sequences. The amino acid diversity was calculated across all positions of Vpr for both the R5 (red) and X4 (blue) sequence populations. While the overall levels of variation tended to be consistent between the two populations, there were notable differences in amino acid diversity between the R5 and X4 populations that persisted across multiple alphabets at positions 28, 37, 55, and 89. Diversity values for amino acid positions at which at least one diversity value was greater than 2 in the full amino acid alphabet are listed in Table 2.

In order to quantitatively assess the meaningful amount of variation occurring within R5- and X4-associated Vpr sequences at the amino acid level, the amino acid diversity for each Vpr position was independently calculated. Furthermore, a series of reduced amino acid alphabets (Murphy-15, Murphy-10, Murphy-8, and Murphy-4) were utilized to assess amino acid diversity [21]. These alphabets hierarchically group amino acids with similar physiochemical properties (Figure 1). As a general trend, the magnitude of the greatest diversity values decreased in concert with reduction of the amino acid alphabet size in both the R5 and X4 Vpr populations. However, the most diverse positions tended to be robustly persistent against the reduction in alphabet size for all alphabets except the highly generalized Murphy-4 alphabet (Figure 2).

A diversity value of 2 in either the R5 or X4 group, using the standard 20 amino acid alphabet, was used as a threshold to define highly diverse positions. The identified positions were located at residues 28, 37, 41, 45, 55, 77, 84, 85, 89, and 93 (Table 2), spanning all three major helical structures of Vpr. Overall, there was no clear trend for either the X4 or R5 populations to consistently display greater diversity across all positions, and most positions displayed approximately equal diversity in the respective populations. In almost all cases, Vpr amino acid diversity was reduced to less than 2 when using the Murphy-4 alphabet, indicating the strong propensity for amino acid substitutions to be contained within the same physiochemical groups once the group sizes become sufficiently large.

There were, however, noteworthy diversity trends at four positions (28, 37, 55, and 89) that persisted across multiple alphabets. Position 28 displayed considerably greater diversity in X4 Vpr when using the full and Murphy-15 alphabets (4.45 versus 3.37 in both cases), but the difference narrowed considerably using the Murphy-10 and Murphy-8 alphabets (3.18 versus 2.95 in both cases). This difference was primarily due to the R5 population being dominated by three variants (R28, N28, and S28) while the X4 population displayed a more even distribution. Positions 55 and 89 both displayed similar trends, in which the R5 diversity trended higher (and over the diversity level threshold of 2) across all alphabets but Murphy-4. This was largely due to the presence of an E55 variant in the R5 population that was entirely absent among X4 Vpr sequences. Similarly, the differential diversity of position 89 was attributed to variant A89 being more highly conserved in the X4 population, with an A89T substitution having no influence on diversity in the Murphy-4 alphabet. Finally, position 37, which was extremely polymorphic, was unique in that the more diverse group switched with the amino acid alphabet utilized. While the X4 group appeared to be more diverse using the standard 20 amino acid alphabets, R5 diversity was greater for each of the four reduced alphabets. At position 37, the most common X4 variants L37, V37, I37, and M37, which contributed to the diversity at this position, collapsed into one group in the Murphy-15 alphabet, while common R5 variants such as P37, S37, and E37 persisted in distinct amino acid groups as the alphabet was reduced.

### 3.2. Divergence in Amino Acid Utilization between X4 and R5 Vpr Is Highly Consistent across Reduced Amino Acid Alphabets

**Figure 3:**
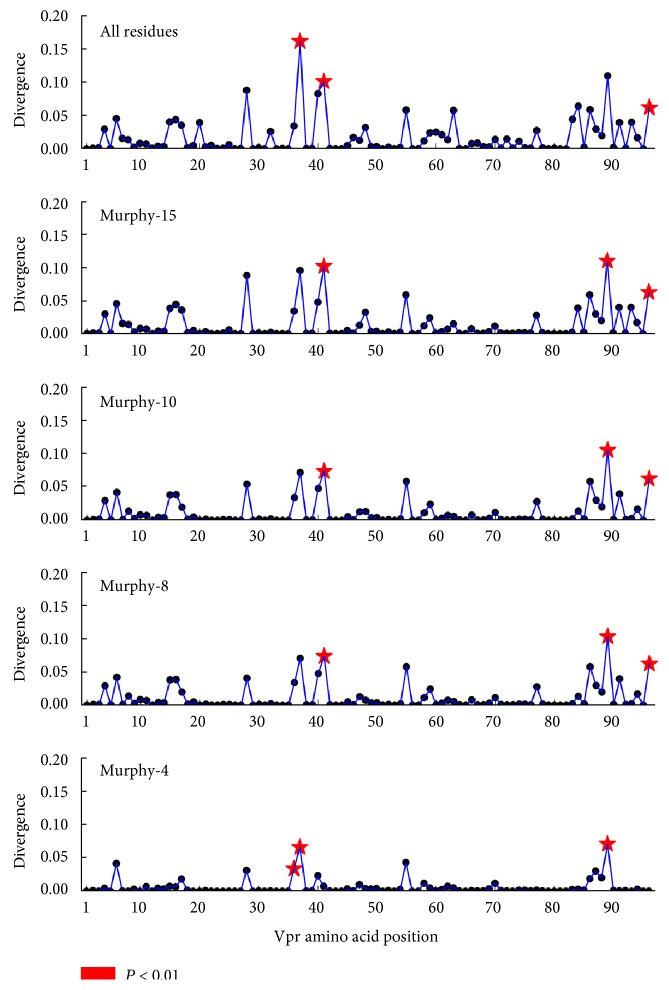
Jensen-Shannon divergence identifies positions with differential amino acid profiles between R5 and X4 Vpr sequences. The Jensen-Shannon divergence between R5 and X4 Vpr sequences was computed for each amino acid position and plotted with a black circle. Statistically divergent positions, as determined by a one-tailed Monte Carlo permutation test (1000 iterations), are indicated by a red star (*P* < 0.05). This methodology was repeated for each of the reduced alphabets, Murphy-15, Murphy-10, Murphy-8, and Murphy-4.

Jensen-Shannon divergence was calculated between amino acid distributions at each position in R5- and X4-associated Vpr sequences (Figure 3), using both the standard amino acid alphabet and reduced alphabets (Figure 1). Jensen-Shannon divergence identifies positions with differential amino acid profiles between R5 and X4 Vpr sequences. Statistically divergent positions (*P* < 0.01) were highly consistent across all alphabets, indicating that the amino acid variation driving Jensen-Shannon divergence was maintained across amino acid categories with increasingly generalized physiochemical properties. Vpr positions 36, 37, 41, 89, and 96 were identified in multiple levels of analysis (Table 3). These positions are concentrated near the second alpha helix of Vpr (36, 37, and 41) and the C-terminal domain (89 and 96).

### 3.3. Amino Acid Substitution Biases in Vpr Are Associated with Coreceptor Genotype

**Figure 4:**
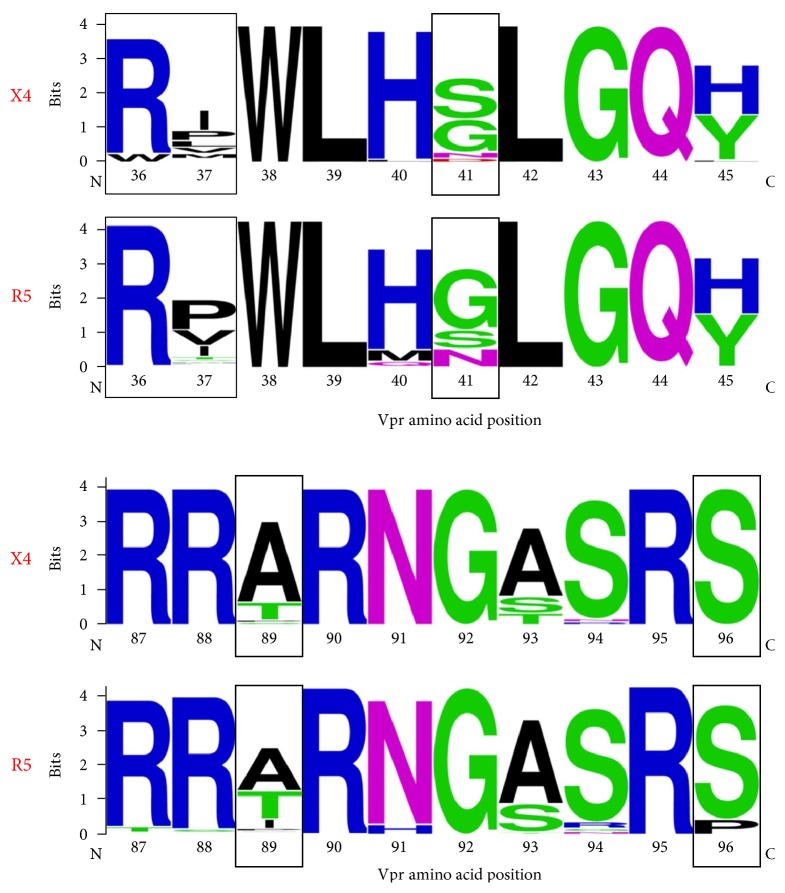
Sequence logos for Vpr regions 36–45 and 87–96. Both regions within the Vpr amino acid sequence included several peaks in amino acid diversity across multiple alphabets. They also contained all positions (36, 37, 41, 89, and 96) found to be statistically divergent using Jensen-Shannon divergence (boxed sequence logos).

Sequence logos were utilized to visualize the two regions of HIV-1 Vpr where amino acid positions displayed significant Jensen-Shannon divergence between X4 and R5 Vpr sequences (Figure 4). In these analyses, Vpr regions 36–45 and 87–96 were selected to encompass all five positions with statistically significant Jensen-Shannon divergence scores in at least one alphabet (Table 3). Positions 37, 41, and 89 displayed consistently high and statistically significant Jensen-Shannon divergence. In accordance with large Jensen-Shannon divergence, positions 37 and 41 both featured considerable shifts in amino acid frequencies and differences in consensus between the R5 and X4 populations, with R5 featuring I37/S41 and X4 featuring P37/G41. In contrast, position 45 was highly polymorphic (H45 and Y45 and a diversity greater than 2 regardless of alphabet) but was not divergent with respect to predicted coreceptor phenotype. Although significant divergence was observed in the Murphy-4 alphabet at position 36 (with the introduction of W36 in the X4 sequences), the consensus at this position (R36) was the same in X4 and R5 sequences. The significant coreceptor phenotype-dependent divergence and consensus changes at positions 37 and 41 provide some evidence for the coordinated or parallel evolution of coding sequences in *vpr* and the V3 portion of *env*.

Similar changes, however, were not observed in conjunction with significant divergence at positions in the C-terminal region of Vpr. At position 89, significant Jensen-Shannon divergence was not accompanied by a consensus shift, although a trend toward a shift from T89 in the X4 sequences to A89 in the R5 sequences was evident. At position 96, the consensus remained S96 even with significant divergence in four of the five alphabets (Table 3). In another example of the independence between divergence and consensus, position 77 displayed a consensus difference between the X4 (Q77) and R5 (R77) populations despite the absence of Jensen-Shannon divergence at this position (data not shown). These results may reflect the lesser importance of specific residues in the C-terminal portion of Vpr with respect to coreceptor usage.

## 4. Conclusions

The role that genetic variation beyond the HIV-1 envelope plays in viral tropism and replication remains a largely unexplored domain. We hypothesized that Vpr would be subject to differential sequence variation as a consequence of cell type-specific roles played by Vpr during replication in a target cell determined by viral coreceptor usage. HIV-1 Vpr has been shown to exist in cell-associated, virion-associated, and virus-free extracellular forms, implicating the protein in multiple aspects of the HIV-1 life cycle [30]. In particular, the virion-associated form of Vpr points to a role for the protein in the early stages of HIV-1 entry and infection, perhaps in the later stages of the viral life cycle as progeny virions are assembled, and with respect to the structural integrity of the final infectious virion, at least with virus produced within cells of the monocyte-macrophage lineage [31]. Vpr associated with virions enables the translocation of the preintegration complex (PIC) into the nucleus of cells such as macrophages [32]. As such, there is also a strong requirement for Vpr during HIV-1 replication in monocytes-macrophages, but expendability in T lymphocytes [10, 33]. In these varied roles, Vpr might be subjected to cell-dependent pressures that would impact the emergence of variants dependent on coreceptor usage and, by extension, host cell type.

The combined use of diversity and divergence analysis allowed us not only to survey the total amount of variation in Vpr but also to test the hypothesis that variation is different between coreceptor-defined sequence groups at specific positions. As such, this methodology allows us to independently assess differences at each amino acid position that may be under selection, a result not afforded by a standard phylogenetic analysis. Due to the presence of high diversity at many amino acid positions throughout Vpr (Figure 2), observation of consensus changes may not always be statistically meaningful with respect to Jensen-Shannon divergence. Alternatively, it is also possible for significantly divergent positions to not demonstrate a consensus change in Vpr amino acid usage between R5 and X4. This is because a change in the relative presence of variants in a polymorphic position, although an indicative of a consensus switch, may fall within the limits of uncertainty if the magnitude of the change is modest. In order to better focus our understanding of amino acid substitutions on potentially functional changes, we expanded our analysis to include a series of reduced amino acid alphabets. While larger alphabets preserve more information from the original amino acid sequence, they can also overemphasize variation among amino acids with similar physiochemical properties. Alternatively, although reduced alphabets exclude potentially meaningful information contained in the original sequence, they maintain differences between physiochemically disparate residues.

The functional impact of the specific amino acid substitutions identified in this study is difficult to infer precisely in the absence of specific mutagenic studies. Nevertheless, positions with differential amino acid occupancy in X4 and R5 sequences were found in regions of Vpr functionally associated with viral packaging, G_2_ arrest, nuclear localization, and alterations in disease severity or progression [34–37]. Of the specific positions observed in this study, positions 36 and 37 are regarded as essential for Vpr oligomerization [38] while position 89 is possibly correlated with clinical manifestations of HIV-1 disease [39]. Structurally, both glycine and proline substitutions may play large roles in altering Vpr secondary structure, especially in alpha helices. Position 37, which is occupied by a proline in consensus R5 Vpr, could potentially lead to the formation of a kink and unwinding of the alpha helix at that position. This effect could be further propagated at position 41 by the presence of a glycine residue, leading to the loss of the helical conformation of helix-2. The C-terminal domain, which includes the divergent position 96, is regarded to play an important role in protein stability [40]. Furthermore, position S96 is often phosphorylated and involved in inducing apoptosis, mitochondrial depolarization, nuclear localization, and transduction of cells by Vpr. While not a statistically significant change with regard to Jensen-Shannon divergence, position 77 showed high diversity in both the R5 and X4 groups, and the consensus sequences both indicated a Q versus R polymorphism at this position. In clinical studies comparing HIV-1 progressor and nonprogressor patients, an R77Q variation was correlated with slower disease progression (long-term nonprogression) [36]. In vitro studies of Vpr demonstrated that the R77Q mutation attenuates Vpr in its ability to induce apoptosis in T lymphocytes [36, 41]. Notably, while position 77 of Vpr is part of the third *α*-helix, the region spanning positions 74–78 exhibits a less strictly defined *α*-helix conformation [7].

In parallel studies of Vpr, we showed that four amino acids at three positions within the primary sequence of Vpr (I37, N41, S41, and A55) were associated with significant differences in patient neuropsychological status [42]. Amino acids N41 and A55 were linked to significant increases in patient neurocognitive deficits, while I37 and S41 were associated with improvements in neurocognitive abilities. The demonstration that positions 37 and 41 are specifically linked to both coreceptor usage and neuropathogenesis is interesting in light of (i) the role of the HIV-1-infected macrophage (infected by an R5 virus) as a “Trojan Horse” that carries HIV-1 into the brain [43, 44] and (ii) the importance of coreceptor usage in HIV-1-associated neuropathogenesis [45–47].

These results suggest that coordinated evolution takes place between Vpr and genetic determinants of viral coreceptor usage. These findings potentially add new dimensions to the already complex and multifaceted involvement of Vpr in HIV-1 infection and associated disease. In HIV-1-infected T lymphocytes and macrophages, coselection of Vpr with coreceptor phenotype may contribute to immunopathogenesis associated with productive infection, as well as the establishment and maintenance of long-lived viral reservoirs even in the face of highly effective antiretroviral therapies. In the brain, coevolution of Vpr with the predominant R5 phenotype of viruses that infect the brain may better position Vpr as a contributor to HIV-1-associated neuropathogenesis. Finally, these studies, as do our previous studies of Tat and LTR coevolution with gp120, suggest the distinct possibility that other viral proteins also evolve in conjunction with viral coreceptor usage.

## Figures and Tables

**Table 1:** Identification of HIV-1 Vpr sequences colinear to Env-V3 sequences defined as R5 or X4. A total of 1010 Vpr sequences were classified according to coreceptor utilization using WebPSSM [19].

Source	CCR5	CXCR4
LANL	949	32
CARES	20	9
Total	969	41

**Table 2:** Diversity values at amino acid positions in Vpr. Each position in the table had at least one diversity value greater than 2 in the full amino acid alphabet. A diversity value greater than 2 (bold) indicates a diverse amino acid position.

Position	Coreceptor	All residues	Murphy-15	Murphy-10	Murphy-8	Murphy-4
28	X4	**4.45**	**4.45**	**3.18**	**3.18**	1.99
	R5	**3.37**	**3.37**	**2.95**	**2.95**	1.76
37	X4	**5.46**	**2.27**	**2.27**	**2.27**	1.94
	R5	**4.97**	**3.14**	**2.93**	**2.92**	**2.16**
41	X4	**2.88**	**2.88**	**2.65**	**2.65**	1.45
	R5	**2.63**	**2.63**	**2.55**	**2.55**	1.64
45	X4	**2.20**	**2.20**	**2.20**	**2.20**	**2.20**
	R5	**2.14**	**2.14**	**2.14**	**2.14**	**2.08**
55	X4	1.87	1.87	1.87	1.87	1.00
	R5	**2.78**	**2.76**	**2.74**	**2.74**	1.38
77	X4	**2.07**	**2.07**	**2.07**	**2.07**	1.00
	R5	**2.31**	**2.31**	**2.31**	**2.31**	1.02
84	X4	**2.94**	**2.20**	**2.20**	**2.20**	**2.20**
	R5	**2.97**	**2.55**	**2.38**	**2.38**	**2.15**
85	X4	**2.71**	**2.71**	**2.71**	**2.71**	1.78
	R5	**2.68**	**2.66**	**2.66**	**2.66**	1.68
89	X4	1.97	1.97	1.83	1.83	1.00
	R5	**3.42**	**3.24**	**3.06**	**3.02**	1.50
93	X4	**2.23**	**2.23**	1.83	1.83	1.00
	R5	1.94	1.94	1.82	1.82	1.00

**Table 3:** HIV-1 Vpr positions with high Jensen-Shannon divergence between R5- and X4-associated sequences. The calculated Jensen-Shannon divergence, along with the associated *P* value in parentheses (in italics when *P* < 0.01), is displayed for positions 36, 37, 41, 89, and 96.

Position	All residues	Murphy-15	Murphy-10	Murphy-8	Murphy-4
36	**0.0346**	**0.0341**	**0.0341**	**0.0341**	**0.0331**
	(0.0367)	(0.0205)	(0.0143)	(0.0271)	*(0.0068)*
37	**0.1612**	**0.0950**	**0.0715**	**0.0709**	**0.0656**
	*(0.0053)*	(0.0322)	(0.0555)	(0.0389)	*(0.0026)*
41	**0.1012**	**0.1012**	**0.0735**	**0.0735**	**0.0072**
	*(0.0013)*	*(0.0005)*	*(0.0024)*	*(0.0034)*	(0.4613)
89	**0.1094**	**0.1094**	**0.1048**	**0.1034**	**0.0704**
	(0.0103)	*(0.0029)*	*(0.0027)*	*(0.0013)*	*(0.0034)*
96	**0.0623**	**0.0623**	**0.0623**	**0.0623**	**0.0000**
	*(0.0051)*	*(0.0029)*	*(0.0030)*	*(0.0030)*	(0.5005)
